# Acoustic Devices for Particle and Cell Manipulation and Sensing

**DOI:** 10.3390/s140814806

**Published:** 2014-08-13

**Authors:** Yongqiang Qiu, Han Wang, Christine E. M. Demore, David A. Hughes, Peter Glynne-Jones, Sylvia Gebhardt, Aleksandrs Bolhovitins, Romans Poltarjonoks, Kees Weijer, Andreas Schönecker, Martyn Hill, Sandy Cochran

**Affiliations:** 1 Institute for Medical Science and Technology, University of Dundee, 1 Wurzburg Loan, Dundee DD2 1FD, UK; E-Mails: y.qiu@dundee.ac.uk (Y.Q.); h.y.wang@dundee.ac.uk (H.W.); c.demore@dundee.ac.uk (C.E.M.D.); a.bolhovitins@dundee.ac.uk (A.B.); r.poltarjonoks@dundee.ac.uk (R.P.); 2 School of Engineering and Computing, University of the West of Scotland, Paisley, PA1 2BE, UK; E-Mail: david.hughes@uws.ac.uk; 3 Faculty of Engineering and the Environment, University of Southampton, Southampton, SO17 1BJ, UK; E-Mails: p.glynne-jones@soton.ac.uk (P.G.-J.); m.hill@soton.ac.uk (M.H.); 4 Smart Materials and Systems, Fraunhofer Institute for Ceramic Technology and Systems, Winterbergstrasse 28, 01277 Dresden, Germany; E-Mails: sylvia.gebhardt@ikts.fraunhofer.de (S.G.); Andreas.Schoenecker@ikts.fraunhofer.de (A.S.); 5 Cell and Developmental Biology, College of Life Sciences, University of Dundee, Dundee, DD1 4HN, UK; E‐Mail: c.j.weijer@dundee.ac.uk

**Keywords:** ultrasonic manipulation, high frequency, array, piezocrystals, screen-printing

## Abstract

An emerging demand for the precise manipulation of cells and particles for applications in cell biology and analytical chemistry has driven rapid development of ultrasonic manipulation technology. Compared to the other manipulation technologies, such as magnetic tweezing, dielectrophoresis and optical tweezing, ultrasonic manipulation has shown potential in a variety of applications, with its advantages of versatile, inexpensive and easy integration into microfluidic systems, maintenance of cell viability, and generation of sufficient forces to handle particles, cells and their agglomerates. This article briefly reviews current practice and reports our development of various ultrasonic standing wave manipulation devices, including simple devices integrated with high frequency (>20 MHz) ultrasonic transducers for the investigation of biological cells and complex ultrasonic transducer array systems to explore the feasibility of electronically controlled 2-D and 3-D manipulation. Piezoelectric and passive materials, fabrication techniques, characterization methods and possible applications are discussed. The behavior and performance of the devices have been investigated and predicted with computer simulations, and verified experimentally. Issues met during development are highlighted and discussed. To assist long term practical adoption, approaches to low-cost, wafer level batch-production and commercialization potential are also addressed.

## 1. Introduction

In recent decades, precise control of bioparticles, biomolecules and biological cells has become increasingly important in life sciences and medicine, with applications emerging in fields such as biochemical analysis, cell separation and sorting, study of cell mechanisms, and tissue engineering. Following this demand, particle manipulation technologies have been developing actively.

One existing approach is to utilize contact-mediated forces applied to targets directly by mechanical tools, e.g., micropipettes [1], atomic force microscopes [2] and micro-grippers [3]. However, direct mechanical intervention can cause problems with mechanical damage to the fragile structure of cells or tissues, and difficulty in handling large numbers of targets. Contactless micromanipulation techniques, such as magnetic tweezing (MT) [4], dielectrophoresis (DEP) [5], optical tweezing (OT) [6], and ultrasonic manipulation (USM) [7] therefore have significant advantages, creating moderate forces to drive particles towards equilibrium states at local potential energy minima without any direct, physical contact. Besides individual manipulation techniques, attention has also been given to combining multiple contactless techniques in single systems, taking advantage of the particular properties of each technique, e.g., integrating USM and DEP [8], and USM and OT [9,10] to achieve precise manipulation of individual cells whilst simultaneously maintaining high throughput.

Generally, all the contactless micromanipulation techniques provide relatively high ability to select specific types of particles as the forces they generate depend strongly on the mechanical properties of the particles and surrounding media, *i.e.*, magnetic susceptibilities in MT, dielectric constants in DEP, refractive indices in OT, and densities and compressibilities in USM. Table 1 outlines qualitatively the capabilities of the contactless micromanipulation techniques, in terms of particle sizes and numbers that can be handled, the typical range of forces that can be produced or measured, preparation of particles such as labelling or seeding required before operation, particle contrast, range of acceptable media, spatial precision, range of the operation field, biocompatibility and system integration.

One critical requirement of MT is that the particle should have either an induced or permanent magnetization. Only two types of cells are naturally magnetic, *i.e.*, red blood cells and magnetotactic bacteria [11]. Therefore, other cells have to be labelled or seeded externally or internally with paramagnetic [12] or magnetic particles [13]. Hence, magnetic tweezers are usually used for manipulation of non-biological objects.

The advantages of using DEP compared with USM for micromanipulation are its reasonable precision and the relative ease of individual particle manipulation. However, to achieve these capabilities, a very small distance between the electrodes is required to provide the necessary gradients in the electric field, which reduces the dimensions of the field of operation, increases the risk of clogging, and brings more difficulties in fabrication. Moreover, conventional cell culture media show good electric contrast and hence their high conductivities may cause significant Joule heating [22], raising difficulties in long-term handling of cells. Researchers have also suggested that electric fields may potentially directly interact with cells via voltage-gated ion channels [42], and both high strength electric fields and low DEP-relevant frequencies affect cell physiology [43].

The main advantages of OT over USM are its high spatial precision and the ability to manipulate nanometre-sized particles and measure forces in the pN range [23,25]. However, in biological applications, the high energy of focused lasers has often induced local heating of the media and photodamage of cells, hence reducing measurement sensitivity and cell viability [17]. Furthermore, the complicated instrumentation brings difficulty in miniaturization and integration with other systems, especially for more complex manipulation applications.

Of the contactless techniques, USM offers several potential advantages. These include ease of integration into microfluidic systems at low cost, maintenance of cell viability, and generation of forces of the amplitudes necessary to handle particles and cells with dimensions up to a few tens of microns and agglomerates of hundreds or thousands of particles. With these advantages, several biological and medical applications employing ultrasonic standing waves have been demonstrated, including cell filtration, washing and sorting [44–49], cell patterning and immobilization [35,39,50], culturing and proliferation of cells in suspension [51,52], capture and accumulation of microbubbles at a target site [53], sensitivity improvement of biosensors and bioassays [54–57], and efficiency enhancement of *in vitro* sonoporation [58,59]. Existing USM devices have valuable capabilities but are limited in terms of forces that can be produced and measured, have constrained trapping sites due to enclosure resonances and device geometries, and involve fabrication complexity which negatively affects progress towards mass production and commercialization.

The limitations that have been outlined have motived the research reported here. Firstly, two approaches to increase the force amplitude have been investigated with the aim of exploring the feasibility of cell biology applications studies of cell motility, by: (1) increasing the operating frequencies of the devices and (2) increasing the acoustic pressure gradients. Secondly, the integration of ultrasonic transducer arrays in USM devices has been investigated with the aim of overcoming the reliance on device resonance limitations imposed by geometry and to open avenues to extend precise manipulation to multiple dimensions. Finally, several well established fabrication techniques from other domains of technology have been explored with the aim to reduce the manufacturing complexities of ultrasonic manipulation devices to move towards mass production. In this paper, all reported USM devices and the related discussion are those in which the nodal planes are parallel to the piezoelectric transducer [60,61], rather than surface acoustic wave (SAW) based devices and other configurations which can excite other resonances.

## 2. Theory

**Figure 1.**
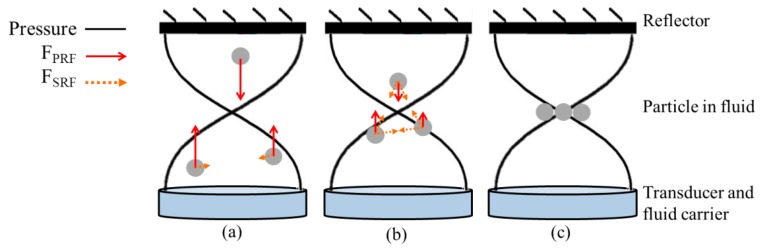
Schematic diagrams of *F_PRF_* (red arrows) and *F_SRF_* (brown dotted arrows) on three stiff and heavy spheres in a fluid subject to an ideal 1-D US field, (**a**) particles moved towards pressure nodal plane by large *F_PRF_*; (**b**) *F_PRF_* decreases and *F_SRF_* increases during the movement; and (**c**) an agglomerate is formed and trapped in a pressure nodal plane. The lengths of the arrows are adjusted for clarity and omitted in the agglomerate.

Small particles suspended in standing acoustic wave fields experience non-zero time averaged forces produced by gradients in the energy densities in the field and scattering of the field from the particles [16]. In the acoustics literature, the phenomenon has been referred to as acoustic “radiation pressure” [62,63] or “radiation force” [64], although it is closely analogous to the “gradient force” discussed in the OT community [6]. In this paper, in line with acoustics usage, we refer to these forces as radiation forces. Figure 1 shows two main kinds of acoustic radiation forces set up in an ultrasound (US) standing wave (USW) field in a multilayer planar resonator [60,61]: primary radiation force (*F_PRF_*) and secondary radiation force (*F_SRF_*).

*F_PRF_* is caused by the primary US field that is introduced into the medium and drives the particles towards the pressure nodes or antinodes of the USW field. *F_SRF_* is produced by mutual interactions between particles if they are close to each other in an US field. This means that the values of *F_SRF_* are often much smaller than those of *F_PRF_* [48]. The amplitudes of *F_PRF_* acting on small objects in an acoustic field have been explored extensively in the literature [62,63,65]. King first provided an expression for acoustic radiation force on a small, rigid, incompressible spherical object in an ideal (inviscid) fluid, where the wavelength of the acoustic wave is much larger than the radius of the object [62]. Yosioka and Kawasima expanded King's theory to compressible objects and expressed time-averaged radiation force on a sphere in a plane USW field with acoustic energy density, *E_ac_*, and acoustic contrast factor, *Φ*, as [63]:

(1) $$\langle F\rangle =4\pi {\text{ka}}^{3}\Phi \sin(2kx)E_{\text{ac}}$$

(2) $$E_{\text{ac}}=\frac{p^{2}}{4\rho_{f}{c_{f}}^{2}}$$

(3) $$\Phi =\frac{\rho_{s}+\frac{2}{3}(\rho_{s}-\rho_{f})}{2\rho_{s}+\rho_{f}}-\frac{\beta_{s}}{3\beta_{f}}$$

where *a* is the radius of the sphere; *p* is the acoustic pressure amplitude; and *ρ*, *c* and *β* are the mass density, speed of sound, and compressibility of the fluid and the sphere, indicated by subscripts *f* and *s* respectively, The relationship of these three parameters is *β* = 1(1*/ρ*c^2^).

Later, the theory of Yosioka and Kawasima was generalized to the case of arbitrary acoustic fields by Gor'kov [65], expressing the time-averaged force as a function of the gradients of the kinetic energy density, 〈*E_kin_*〉, and potential energy density, 〈*E_pot_*〉, of the acoustic field:

(4) $$\langle F\rangle =\nabla \left(\frac{4\pi a^{3}}{3}\left(\frac{3(\rho_{s}-\rho_{f})}{2\rho_{s}+\rho_{f}}\langle E_{\text{kin}}\rangle -\left(1-\frac{\rho_{f}{c_{f}}^{2}}{\rho_{s}{c_{s}}^{2}}\right)\langle E_{\text{pot}}\rangle \right)\right)$$

〈*E_kin_*〉 and 〈*E_pot_*〉 are related to the acoustic velocity, *u*, and acoustic pressure field, *p*, by [34]:

(5) $$\langle E_{\text{kin}}\rangle =\frac{1}{2}\rho_{f}u^{2}$$

(6) $$\langle E_{\text{pot}}\rangle =\frac{1}{2\rho_{f}{c_{f}}^{2}}p^{2}$$

The theoretical developments of King, Yosioka and Kawasima, and Gor'kov still underpin new developments in USM.

## 3. Design and Modelling

### 3.1. Materials

#### 3.1.1. Piezoelectric Materials

Although piezoelectric ultrasonic transducers can use many different piezoelectric materials as their active elements, e.g., piezoceramics, conventional and high performance relaxor-based piezocrystals, piezocomposites and piezopolymers [66], the basic specifications of multilayer resonator type USM devices and their applications restrict the selection of the piezoelectric materials in practice as listed in Table 2.

LiNbO_3_ (LNO) has a very high *Q_m_*, up to 10^4^, and a high Curie temperature, *T_C_* ≈ 1200 °C. It has been used in ultrasonic devices for high temperature non-destructive testing [67] and high power focused US surgery [68]. As a highly anisotropic crystal, it has distinct piezoelectric properties depending on its orientation. Among all the different possible orientations, Y-36°-cut LNO is attractive because it is quasi-longitudinal and has the highest coupling coefficient, *k_t_* = 0.495, about three times that of a pure longitudinal mode Z-cut. Also, it has a very high resonant frequency constant, of about 3.3 MHz/mm, allowing thicker piezoelectric elements at high frequencies compared to the common lead zirconate titanate (PZT) ceramic. In the authors' experience, these properties make Y-36°-cut LNO a particularly good material for high frequency USM devices. Moreover, the optical transparency of LNO is potentially useful in combining USM and OT in hybrid systems [10].

PZT is the most common piezoelectric material in ultrasonic transducers, and can be processed in bulk, thick-film and thin-film forms, according to application requirements. Bulk PZT is usually mechanically strong, dense and with low porosity [69]. As a trade-off, it is conventionally manufactured relatively thick, e.g., >500 μm and thinning is required to achieve a desired thickness and final shape during transducer fabrication, limiting its application in miniature and complex devices, e.g., micro- and nano-electromechanical systems (MEMS and NEMS). Instead, MEMS/NEMS requiring piezoelectric features <200 μm thick can be fabricated using thick film and thin film technologies. PZT films are usually deposited on substrates and integrated with interconnections. However, if the substrate is similar to or thicker than the deposited PZT film, the effective piezoelectric response is changed because in-plane clamping yields stress within the film [70].

Relaxor-based ferroelectric piezocrystals, e.g., PMN-PT, PIN-PMN-PT, and Mn:PIN-PMN-PT, exhibit much higher piezoelectric constants and electromechanical coupling coefficients than conventional PZT [71,72], e.g., *d_33_* > 1300 pC/N and *k_33_* ≈ 0.9. These piezocrystals are usually implemented in the form of piezocomposites [72] because this form of material exploits the high values of *d_33_* and *k_33_*, allowing for the fact that *k_t_* ≈ 0.5 is similar to that of conventional piezoceramics. Piezocomposites provide a method to further tailor the physical, mechanical and piezoelectric properties of bulk piezoelectric materials by combining them with polymers. In addition, the lossy polymer matrix suppresses ringing and most lateral resonances, leading to higher sensitivity and larger bandwidth in ultrasonic devices [73,74].

#### 3.1.2. Passive Materials

Passive materials also play important roles in multilayer resonator type USM devices, e.g., as acoustic matching and physical support layers, and in forming resonant structures, e.g., carrier and reflector (Figure 1). Matching layers are not always used in the ultrasonic transducers of USM devices because other layers, e.g., US couplant and fluid carrier, are used to couple the piezoelectric material to the working volume, and because their creation adds needlessly to the complexity of device fabrication. Air backing is used for most multilayer USM devices to maximize energy output at the transducer front face [75]. However, as high frequency devices generally have thin piezoelectric elements, physical support layers are required, e.g., a low acoustic impedance (low *Z_ac_*) backing.

The material considerations for reflectors in USM devices include dimensional stability and smooth surfaces. High *Z_ac_* compared to the value for the fluid in the working volume (*Z_ac_* ≈ 1.5 MRayl) is typically used in USM designs. When handling biological cells, the biocompatibility of the material is important if the cells are in direct contact. For carriers and spacers used for constructing fluid chambers and channels, formability and machinability are important concerns; thus materials with acoustic impedances close to water can also be used, e.g., polydimethylsiloxane (PDMS) and polymethyl-methacrylate (PMMA). Besides these common materials, machinable glass ceramic, Macor (Corning, NY, USA) may also be a good choice for carrier, spacer and reflector, because it has similar chemical and acoustical properties to glass, plus good machinability to allow desired thicknesses and complex shaping [76]. Researchers have also demonstrated the feasibility of glass capillaries in USM devices, with advantages in terms of disposability, sterility, simplicity of use and good resonant characteristics [75]. Nevertheless, care must be taken as dimensional variations can lead to small changes in resonant frequency which interact badly with the high *Q_m_* of many USM devices.

### 3.2. Devices for Generation of Large Acoustic Forces

According to Equation (1), two effective means to increase the amplitude of *F_PRF_* on particles in fluid are to increase the wave number, *k*, and the acoustic pressure, *p*. Therefore high frequency ultrasound (HFUS) transducers are preferred to reduce the wavelength and increase the wave number. This can also lead to USWs with small wavelength, thus generating multiple trapping lines even in a small chamber but allowing high precision manipulation. In the same vein, utilization of HFUS (>20 MHz) in SAW based manipulation devices has already been extensively explored for focusing and separation of micron- and nanometer-sized particles [38–40,77]. Moreover, mixing of nanometer-sized particles in nanoliter droplets with SAW based devices of frequencies in GHz range has also been demonstrated [78]. Straightforward ways to increase the acoustic pressure are to increase the electrical input power to the transducer and to use focused US transducers. Increasing input power, however, differentially increases the risk of streaming and of heating. Here, two relevant transducer configurations are reported, planar and curved ultrasonic resonators.

#### 3.2.1. Planar Ultrasonic Resonator

**Figure 2.**
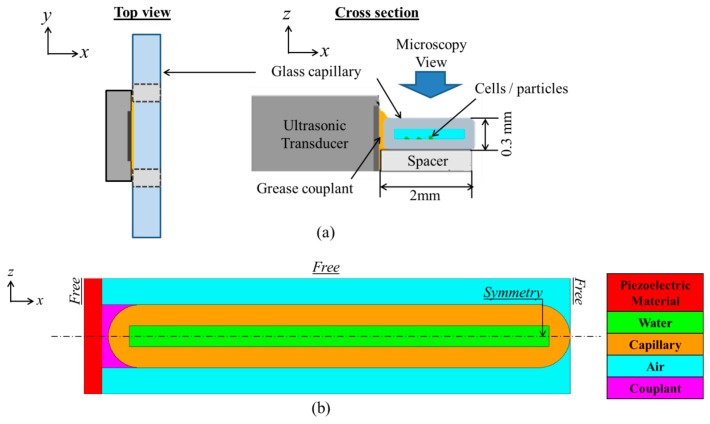
(**a**) Schematic diagram of the planar ultrasonic resonator; and (**b**) PZFlex model of the resonator with assigned materials and boundary conditions.

The planar US resonator, Figure 2a, comprises a Y-36°-cut LNO HFUS transducer of frequency, *f* ≈ 23.8 MHz and a disposable glass capillary (W5010, VitroCom, Mountain Lakes, NJ, USA) with targeted particles or cells inside. The capillary has channel dimensions 100 μm × 2 mm and wall thickness ∼100 μm. A 2-D model of the device was created for finite element analysis (FEA) using the PZFlex code (Weidlinger Associates Ltd., Glasgow, UK), as shown in Figure 2b. In PZFlex, piezoelectric materials are defined by the complete elasto-electric matrix. However, the lack of data for Y-36°-cut LNO brings difficulties; therefore a PZ26 transducer with the same resonant frequency was substituted to predict the device behavior before manufacture. The passive materials were assigned properties already defined within PZFlex libraries.

The capillary was coupled to the transducer with 30 μm thick couplant, required to avoid air in the practical device configuration. The couplant thickness was estimated experimentally and three types were modeled, *i.e.*, medical US couplant (*Z_ac_* ≈ 1.5 MRayl), NDT gel (*Z_ac_* ≈ 3.69 MRayl) and a dummy couplant (*Z_dummy_* ≈ 19 MRayl, calculated by $Z_{\text{dummy}}=\sqrt{Z_{\text{pz}26}\times Z_{\text{glass}}}$). Symmetry boundary conditions were assigned to reduce the size of the model and runtime. The model was driven with one sinusoidal cycle at twice the maximum frequency of interest. The mesh size of the model was set to λ_w_/20, where *λ_w_* is the wavelength in water at the frequency of interest. The runtime allowed the US to make 10 round trips across the water channel. After coupling to the capillary, with medical US couplant, the electrical impedance and pressure response spectra of the transducer were extracted. The frequencies at which peak pressure responses occurred were selected to observe the pressure and velocity fields.

**Figure 3.**
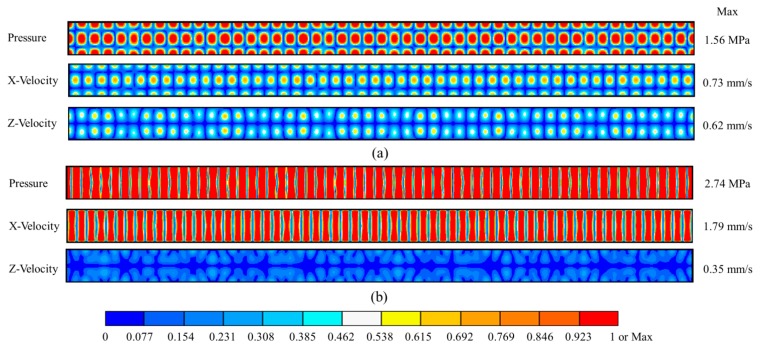
Normalized pressure and velocity fields in the water channel of the capillary at (**a**) 22.67 MHz and (**b**) 23.46 MHz with 1 V_p_ input.

**Figure 4.**
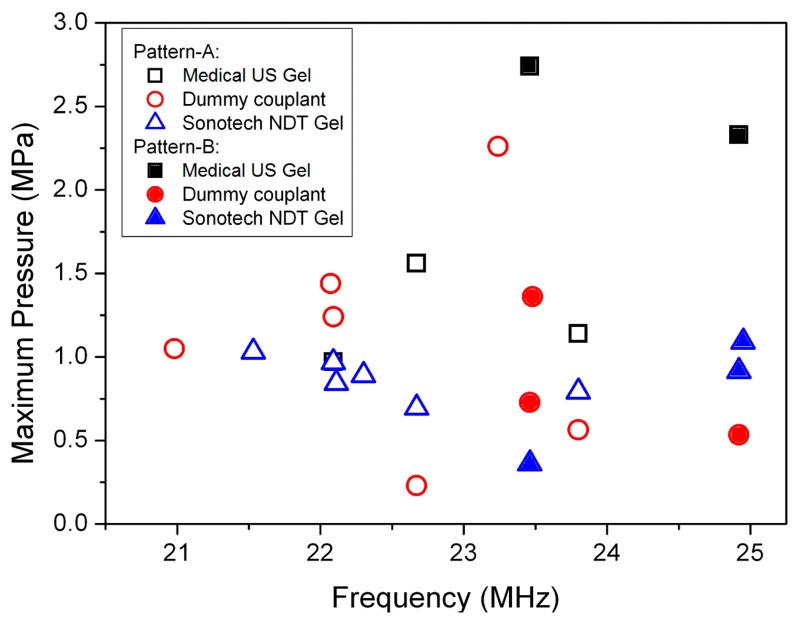
Maximum pressure generated in the capillary water channel with different couplant materials at different values of *f* with 1V_p_ input.

Figure 3 shows two typical USW patterns, *i.e.*, pressure and velocity mode-shapes, formed at different frequencies, due to the dominant effects of capillary geometry on the resonant modes. Pattern A occurs when a USW or quasi-USW is formed parallel to both *X* and *Z* axes at *f* = 22.67 MHz, Figure 3a. Pattern B occurs when a USW or quasi-USW is formed parallel to the *X* axis only, at *f* = 23.46 MHz, Figure 3b. Similar mode-shapes were also obtained with NDT gel and dummy couplants. The maximum pressures generated in the water channel with different couplants at different frequencies are shown in Figure 4. The frequencies to form Pattern B with the three different couplants are all near *f* = 23.5 MHz and *f* = 24.9 MHz. The field patterns are extremely sensitive to frequency, e.g., with only ∼0.3 MHz frequency difference between two patterns at *f* = 23.46 and *f* = 23.80 MHz. Such high sensitivity to *f* must be taken into account during practical experiments.

US transmission and reflection at media interfaces strongly depend on the values of *Z_ac_* for the media. Thus, the pressure amplitude with NDT gel and the dummy couplant should be larger than with medical US couplant. However, this is not evident in Figure 4. The reasons are thought to be (1) the non-optimized thickness of the coupling layers; (2) the rounded side-wall of the glass capillary; and (3) the frequency sensitivity of the peak pressure making it easy to miss the highest response because of the quantized frequency step, *Δf*, in FEA. In any case, the pressure responses in the water channel at the selected frequencies are still relatively high, >0.25 MPa/V_p_.

#### 3.2.2. Curved Ultrasonic Resonator

**Figure 5.**
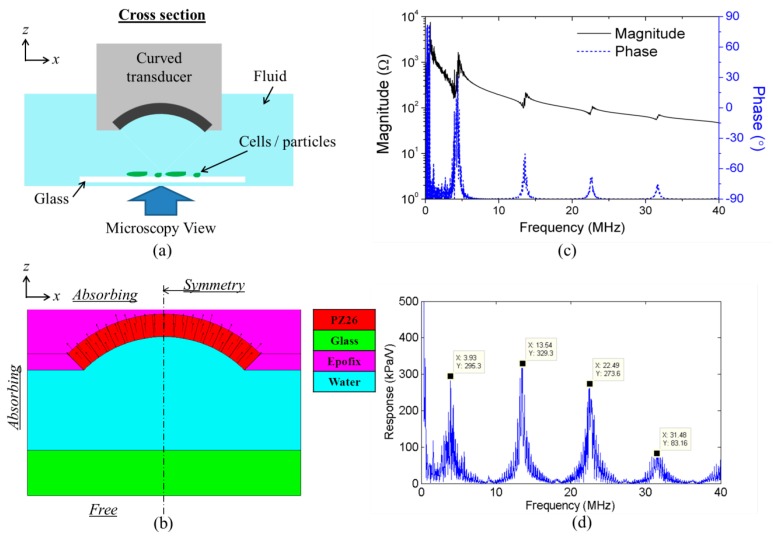
(**a**) Schematic diagram of the curved ultrasonic resonator; (**b**) PZFlex model with assigned materials and boundary conditions; (**c**) impedance spectrum of the resonator; and (**d**) acoustic pressure response spectrum at the focal point of the transducer.

Focused ultrasound trapping has been demonstrated in the literature [35], however, the work used ultrahigh frequencies, e.g., 200 MHz, thus *λ_w_* ≈ *a*, giving rise to gradient-forces across small objects analogous to the physics of OT. Particle and cell trapping have also been demonstrated by creating an energy potential well with the counter-propagating US beams transmitted from two collimated focused US transducers, and the movement of the trapping site has been realized by tuning the frequency and location of one of the focused transducers [32]. Similarly to this setup, USM devices with annular chambers created by multiple of counter-propagating transducers demonstrated much more control dexterity of the trapping sites by tuning the phase of these transducers [33,41]. For the curved US resonator reported here, Figure 5a, *F**_PRF_* was generated in an US field where *λ_w_* ≫ *a*, using a curved PZ26 transducer to transmit a focused US beam towards a glass reflector, thus forming a quasi-USW. By moving the position of the curved US transducer laterally from the position-fixed reflector, the trapping site can be moved simultaneously. As PZ26 is a hard piezoceramic, the transducer can be driven at harmonics as well as the fundamental frequency.

Again, a model of the resonator was created in PZFlex, including a quarter-ring PZ26 transducer (*OD* = 6 mm and *ID* = 5 mm) mounted in Epofix (Struers Ltd., Catcliffe, Rotherham, UK) loaded with glass microballoon (GMB – K1, 3M United Kingdom PLC, Bracknell, UK), with a water layer and a 1 mm thick glass slide as the reflector, Figure 5b. The piezoelectric and passive materials were assigned properties defined in the PZFlex libraries but, because each volume of piezoelectric in PZFlex can be assigned only one unique poling direction, the radially poled quarter-ring transducer was divided into 18 pieces, each representing a 5° arc, with individual poling directions. The reflector was placed so as the focal point of the transducer field was located at its front surface, with a corresponding symmetry boundary condition used to reduce the model size. Absorbing boundary conditions were assigned on the edges of the Epofix to represent the high scattering and absorption of the GMB-loaded Epofix. A free boundary condition was assigned to represent the air behind the glass. The model was driven with one sinusoidal cycle at a frequency of 45 MHz, ∼10 times the fundamental resonance frequency of the quarter-ring transducer. The mesh size of the model was set as *λ_w_*/15, where *λ_w_* is the wavelength in water at 45 MHz. The run time was set to allow the US to make five round trips from the inner arc center of the quarter-ring transducer to the glass reflector.

**Figure 6.**
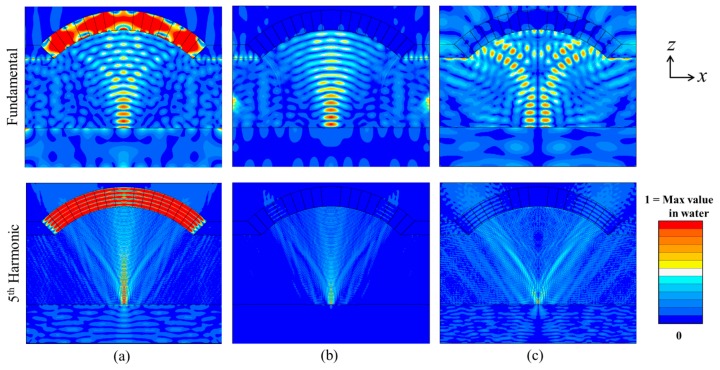
Normalised (**a**) pressure, (**b**) *Z*-velocity and (**c**) *X*-velocity fields of the curved ultrasonic resonator at the fundamental and 5th harmonic resonant frequencies.

The impedance spectrum and pressure response spectrum at the focal point were extracted, with the fundamental and 3rd, 5th and 7th harmonic resonances clear in Figure 5c,d. The normalized pressure and velocity fields at the fundamental and 5th harmonic frequencies were then extracted, Figure 6.

This shows that a quasi-USW was formed in the water, especially clearly near the focal point. The pressure node with *E_pot_* minimum, which is also the *Z*-direction velocity antinode, is about a half wavelength from the glass reflector in the central line, Figure 6a,b.

Therefore, *F_PRF_* will arise on particles already near the middle of the glass slide. Depending on the relationship between *F_PRF_* and the buoyancy and gravity forces, the particles can potentially be lifted and gathered at the *E_pot_* minimum (pressure node) and *E_kin_* maximum (*Z*-velocity anti-node). The trapped particles can then be moved by moving the transducer laterally as demonstrated in literature [32,35]. Because of the transducer geometry, a non‐uniform *X*-direction velocity field is also generated, Figure 6c, located away from the central line at the boundary between the reflector and the water, with a maximum amplitude about a quarter of the maximum amplitude of the *Z*-velocity. This makes it possible that particles at some positions will be pushed away from the central line towards the *X*-direction velocity anti-nodes.

### 3.3. Devices for Precise Manipulation in Multiple Dimensions

**Figure 7.**
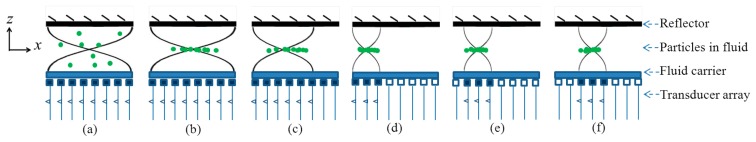
USM with a transducer array integrated into a multilayer resonator: (**a**) USW generated between an US transducer array and a reflector; (**b**) particles in fluid layer move towards pressure nodal plane; (**c, d**) reducing the number of active elements of the array, lateral acoustic energy density gradients move particles above the middle of the active elements; (**e, f**) selecting other active elements moves trapped particles along fluid channel.

In general, precise control over particle position with US has been limited by enclosure resonances and hence the geometry of USM devices [34]. Although efforts have been made to overcome this, most are limited to controlling the particle only in the direction of wave propagation or by mechanically moving the US source [35,79–81]. However, a practical USM field always has variations in directions normal to wave propagation, which generate *F_PRF_* to drive particles towards particular points laterally in the nodal and antinodal planes. By utilizing the variations in *E_kin_* energy density, particle trapping at particular locations within the nodal and antinodal planes has been demonstrated [82–84], however, most of them are lack of control flexibility limited by the use of single US transducer. Thus, with controllable variation of an US field created with US transducer arrays, precise lateral control and better control flexibility can be realized, as shown schematically in Figure 7.

**Figure 8.**
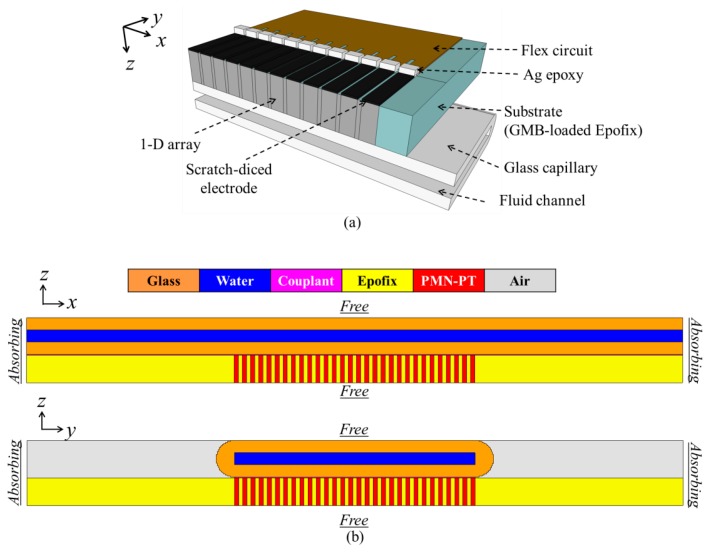
(**a**) Diagram of a quarter of a 1-D ultrasonic transducer array coupled to a glass capillary; (**b**) 2-D PZFlex models of the device in *xz*-plane cross-section, where each PMN-PT pillar indicates one array element, and *yz*-plane, where all 30 PMN-PT pillars are within the outline of one array element.

To demonstrate feasibility of lateral manipulation with an US transducer array, a scratch-diced 1-D ultrasonic transducer array coupled to a glass capillary was studied, Figure 8a. A glass capillary of 300 μm channel and wall thicknesses (W3530, VitroCom) was chosen; the channel thickness corresponded to a 2.5 MHz *λ_w_*/2 resonance so a ∼2.5 MHz 1-D US transducer array was used, based on PMN-PT piezocomposite. Fine-meshed PZFlex models were required to gain sufficiently accurate results; thus, instead of time consuming 3-D models, two 2-D models were created to simulate the device in the *xz*- and *yz*-planes, Figure 8b.

In the models, PMN-PT was assigned properties reported in the literature [71] and other materials were assigned properties defined in the PZFlex libraries. The dimensions of each PMN-PT pillar were 100 × 100 × 450 μm^3^.

**Figure 9.**
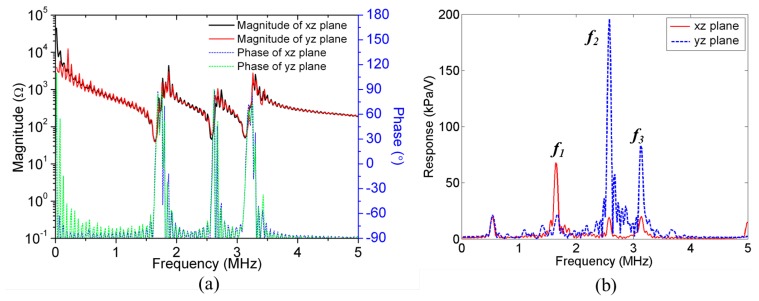
(**a**) Impedance spectra and (**b**) pressure spectra at center point of water layer in *xz*-plane and *yz*-plane models.

**Figure 10.**
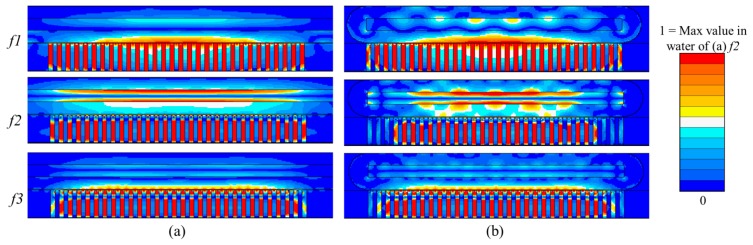
Pressure fields of (**a**) all 30 array elements (*xz*-plane); and (**b**) 30 PMN-PT pillars (*yz*-plane) driven at the peak response frequencies.

Couplant gel 25 μm thick was included between the transducer array and the capillary. A mesh size of 6.25 μm was used for the water and couplant layers and 25 μm for other materials. The model was driven with one sinusoidal cycle at *f* = 5 MHz, approximately twice the frequency of interest. Runtime was set to be sufficient for the US to make 10 round trips across the whole capillary. The impedance spectra and the pressure spectrum at the center of the water layer are shown in Figure 9. Three high pressure peaks are evident at *f_1_* = 1.64 MHz*, f_2_* = 2.58 MHz and *f_3_ =* 3.14 MHz in Figure 9b. These frequencies were selected to obtain the pressure and velocity fields. Figure 10 shows the pressure fields when all 30 array elements (*xz*-plane) and 30 PMN-PT pillars (*yz*-plane) were driven at the peak response frequencies. In the *xz*-plane, the highest USW pressure amplitude with 1 V_p_ input is 0.238 MPa at frequency *f_2_*; all pressure amplitudes were normalized to this. For both the *xz*- and *yz*-planes, there is one pressure node in the water layer near the bottom water channel wall at *f_1_*; *f_2_* sees one pressure node near the central line of the water channel; and *f_3_* sees two pressure nodes, one in the bottom channel wall near the water layer and the other near the center line of the channel.

These results indicate that *f_2_* and *f_3_* are suitable for driving the device at a *λ_w_*/2 resonance. However, because *f_3_* generates a pressure node in the channel wall, it tends to raise a force towards the bottom of the fluid layer. In contrast, *f_2_* generates only a force to push particles away from the bottom of the channel. Also, because the pressure magnitude of the USW is much higher for *f_2_* than *f_3_*, the force resulting from excitation at *f_2_* is generally higher than for *f_3_*. This indicates that *f_2_* is more useful practically in moving dense particles to the middle of the water channel. Similar phenomena can be observed in the *yz*-plane model, but with corrugated fields caused by the capillary geometry in the *y* direction, Figure 10b.

**Figure 11.**
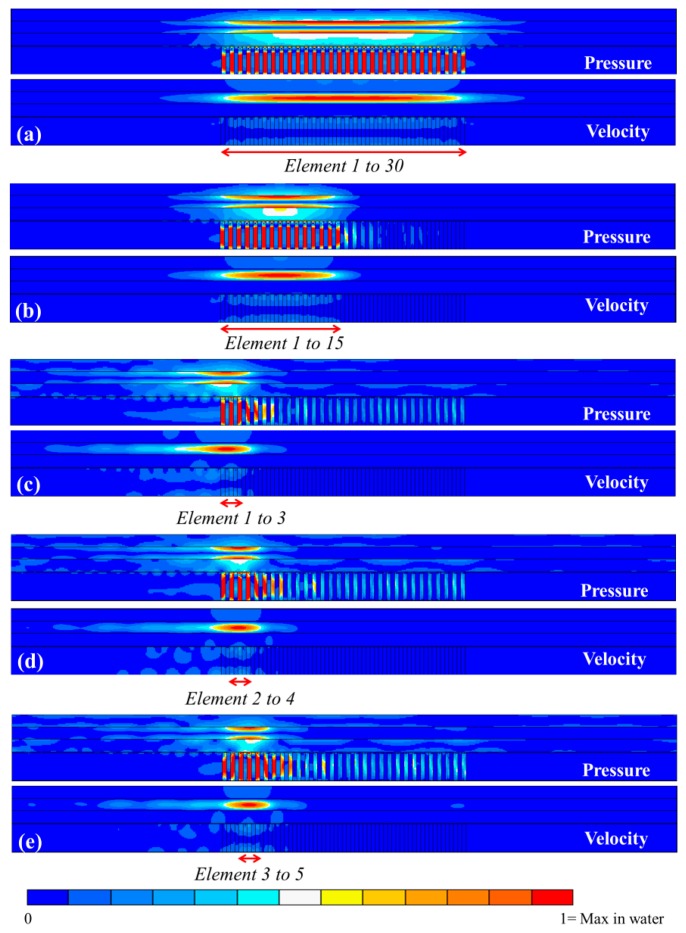
Pressure and velocity distributions of the 1-D array lateral: (**a**) all 30 elements active; (**b**) 15 elements active; and (**c–e**) sets of 3 elements active, shifted along the channel. The elements are driven at *f_2_*. Each field is normalized to its own maximum amplitude in the water. Red arrows and text indicate active elements.

Following this analysis, *f_2_* was applied to the model to investigate pressure and velocity distributions under different conditions: (1) with different numbers of active elements in the array, and (2) altering the active elements along the water channel. Figure 11 shows the pressure and velocity distributions under different conditions in the *xz*-plane.

The amplitude of the USW field is higher when a larger number of elements is activated, simply because of higher acoustic energy transmission. However, amplitudes are similar when different sets of the same number of elements are active, e.g., showing less than 5% difference in Figure 11c–e. In addition, no matter how many elements are active in a continuous set, the pressure minimum is always close to the center line of the channel, and the velocity maximum is always above the middle of the active elements at the center line. Hence, dense particles in water will be driven towards the *E_pot_* density minimum, *i.e.*, the pressure nodal plane close to the center line of the channel. Once the particles reach this plane, there is no further contribution from the *E_pot_* density terms (Equation (4)), and the *E_kin_* density term provides an additional force to drive the particles to the *E_kin_* density maximum, *i.e.*, above the active elements in the channel.

As discussed earlier, the lateral force component caused by *E_kin_* density is typically smaller than the axial component from *E_pot_* density. Therefore, the particles first move rapidly to the vertical central line then slowly agglomerate above the middle of the active elements. When the same number of active elements is shifted along the channel, the change in the position of the *E_kin_* density maximum leads to the trapping site moving along the center line of the channel, as shown in Figure 11c,d.

### 3.4. Devices towards Mass Production

Depending on the desired thickness of the PZT film, a number of fabrication techniques are available. The modified sol-gel method [85] and hydrothermal deposition [86] have been used for thicknesses of 10–30 μm. Aerosol deposition can achieve uniquely high density films, theoretically >95%, with thickness range 1–100 μm [87]. Screen-printing [88] can achieve a large thickness range, typically 10–150 μm. The modified sol-gel process overcomes the thickness limitation of the conventional sol-gel process by incorporating ceramic powder into the chemical solution, thus producing thick films at a relatively low sintering temperature, around 700 °C.

**Figure 12.**
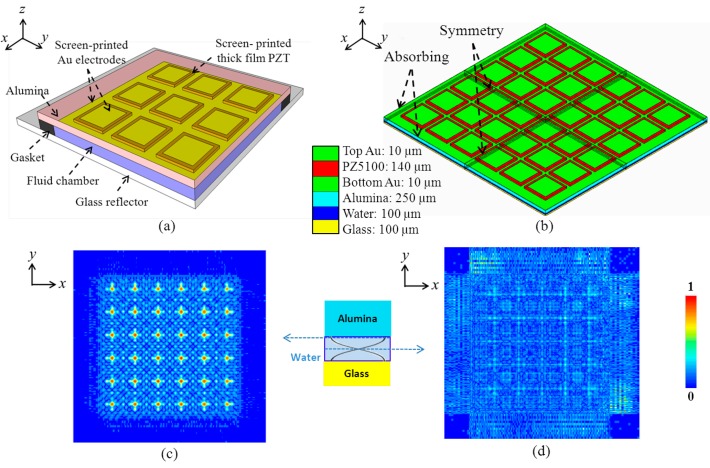
(**a**) Cross-sectional diagram of quarter of a multilayer USM device with a screen-printed thick-film PZT 2-D matrix array; (**b**) 3-D FEA model and layer thicknesses of 2-D matrix thick film array manipulator; (**c**) normalized pressure distribution near the anti-nodal and (**d**) nodal planes in water beneath the alumina substrate of the 2-D array with all 36 elements active. © 2013 IEEE. Reprinted with permission from [91].

The hydrothermal method is able to make piezoelectric monocrystalline and polycrystalline films on a large area at <200 °C [89]. Aerosol deposition has been reported to achieve dense, crack-free ceramic films based on the impact and consolidation phenomenon of ceramic particles at room temperature with a high deposition rate. Patterning can be achieved by successive lift-off process [90]. Compared with other fabrication techniques, screen-printing is a relatively simple way to fabricate patterned films with reasonable quality and excellent reproducibility. Hence, creation of a 2‐D matrix ultrasonic transducer array manipulator, Figure 12a, based on thick film screen printing was used to demonstrate the reduction in fabrication complexity, for translation towards mass production.

A 3-D FEA model was created with 100 μm water thickness, Figure 12b. The element pitch in both dimensions was 2.3 mm and the element dimensions were 2 × 2 mm^2^, with top electrodes of 1.7 × 1.7 mm^2^. As the PZT is built up layer-by-layer in screen printing, the PZT structures have rounded edges. Hence, the dimension of the top electrode on each PZT element was 0.3 mm smaller. The dimensions were also optimized to obtain an electrical impedance magnitude of ∼50 Ω at the fundamental resonance, *f* ≈ 7.5 MHz for ease of electrical impedance matching. The FEA mesh size was ∼*λ_w_*/20 in the z-direction and ∼*λ_w_*/5 in the *x*- and *y*-directions, because of their larger dimensions. The model was excited by a short pulse and oscillation was allowed to decay completely. The mode shape was calculated at the frequency of maximum response. Normalized pressure distributions near the anti-nodal and nodal planes in the water are shown in Figure 12c,d. The width at half the pressure amplitude maximum is approximately 0.4 mm. Because of the lack of overlap of energy gradients because of the larger lateral element dimensions, this design is unable to transport particle agglomerates by switching active array elements as in the 1-D device; an array with a smaller pitch and element size would be required to realize such lateral transportation.

## 4. Experimental Results and Discussion

### 4.1. Planar Ultrasonic Resonator

A 23.3 MHz ultrasonic transducer was fabricated from a 3-inch Y-36°-cut LNO wafer (Roditi International Corporation Ltd, London, UK) with GMB-loaded Epofix as backing using a fabrication process reported elsewhere [76], but was further lapped to obtain a height of 1.1 mm in the elevation direction of the transducer.

**Figure 13.**
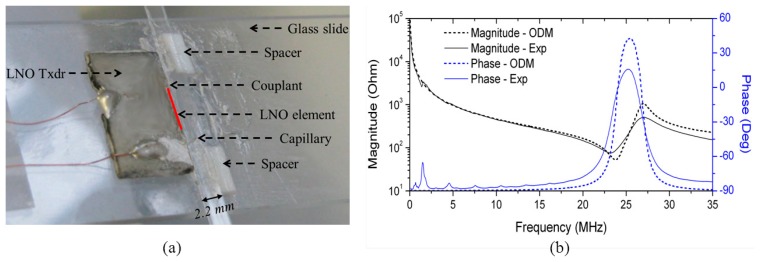
(**a**) Planar ultrasonic resonator; (**b**) the experimental electrical impedance spectrum of the transducer compared to the ODM modelled electrical impedance spectrum of the LNO element.

Two Macor spacers were lapped to a thickness of 0.4 mm and used to lift the capillary into the plane of the transducer. A glass capillary of 0.1 × 2 mm^2^ channel dimension and 0.1 mm wall thickness (W5010, VitroCom) was coupled to the LNO transducer front face with US couplant, as shown in Figure 13a. The impedance spectrum of the transducer was measured in air, and compared to the modelled results of a LNO element of the same dimensions in the practical transducer using one-dimensional model (ODM [92]), as shown in Figure 13b. The experimental result suggests slightly higher damping and a slightly lower resonant frequency because of the GMB-loaded Epofix mounting on the LNO element.

**Figure 14.**
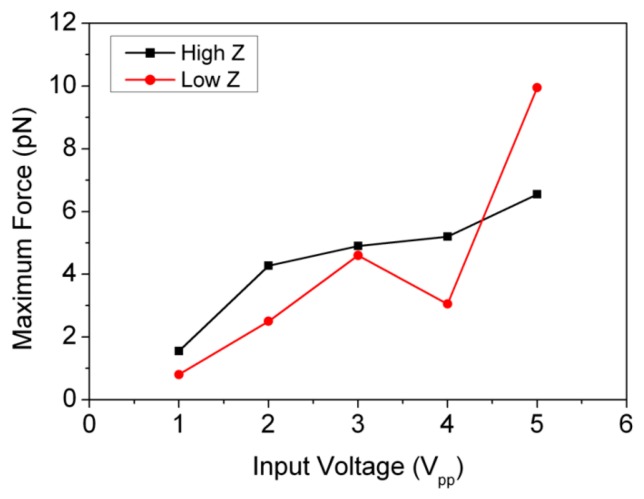
Estimated maximum acoustic radiation forces on Ø10 μm fluorescent polystyrene beads at different input voltages.

An experiment was performed with beads to evaluate the performance before cell experiments. A mixture of Ø10 μm fluorescent polystyrene beads (Polysciences Inc., Warrington, PA, USA) in degassed water (bead concentration ∼1.5 × 10^6^ particles/mL) was drawn into the capillary by “capillary action”. The motion of the beads was recorded with an epi-fluorescence microscope. The operating frequency of the device was switched between two resonant frequencies, 24.96 MHz and 27.00 MHz, thus rearranging the trapped beads after each switch. Two different couplants with low (*Z_ac_* ≈ 1.5 MRayl) and high (*Z_ac_* ≈ 3 MRayl) acoustic impedances were used. The maximum acoustic radiation forces on the beads were estimated from the viscous drag forces calculated from measured velocities [34] at different input voltages, Figure 14. Input voltage was limited to 5 V_pp_ because strong streaming effects were found at higher amplitudes.

The maximum force is around 10 pN for 10 V_pp_ input, corresponding to a pressure of 0.048 MPa, calculated from Equation (1). This is relatively low compared with the FEA results, >0.5 MPa/V_p_, with substitution of a PZ26 transducer in the device. The reasons are thought to be two-fold: (1) the measured output pressure from the LNO transducer is degraded approximately –3 dB at the operating frequency *f* = 24.9 MHz, which is mismatched with the 23.3 MHz resonance of the transducer, and (2) the device is highly sensitive to driving frequency, thus leading to the frequency of the highest response being missed during the experiment. Replacing the capillary, small frequency shifts between 24.6 and 25.2 MHz achieved best device performance because of unavoidably different couplant thicknesses and small variation in capillary dimensions. This therefore brought difficulty in the selection of operating frequency, resulting in weak acoustic radiation.

Cell manipulation evaluation was performed with fluorescence-stained *Dictyostelium discoideum* (Dicty). Details of culturing, harvesting and preparation have been reported elsewhere [93]. Dicty cells were used in buffer fluid with a concentration of ∼10^6^ cells/mL. The transducer was driven with a 24.6 MHz sinusoidal CW signal at 10 V_pp_. Image sequences of the cells were recorded using Micro‐Manager (Vale Lab, UCSF, San Francisco, CA, USA) microscopy software with and without the presence of USWs across the capillary, for 30 min each. Dicty cell motion was tracked and plotted as polar plots, with the starting point of each cell track shifted to a common origin [93].

In the absence of USWs, cells moved fully randomly, producing tracks migrating away from the origin approximately uniformly in all directions. In the presence of USWs, the polar plot exhibited a slight bias along the pressure nodal lines, perpendicular to the direction of US propagation. Given the noted weak acoustic radiation, it is expected that more substantial effects may be realized by further optimization of the device design, particularly through automatic frequency tracking, which may allow cell motility forces to be evaluated, e.g., on the nN-scale required to influence adherent migration.

### 4.2. Curved Ultrasonic Resonators

**Figure 15.**
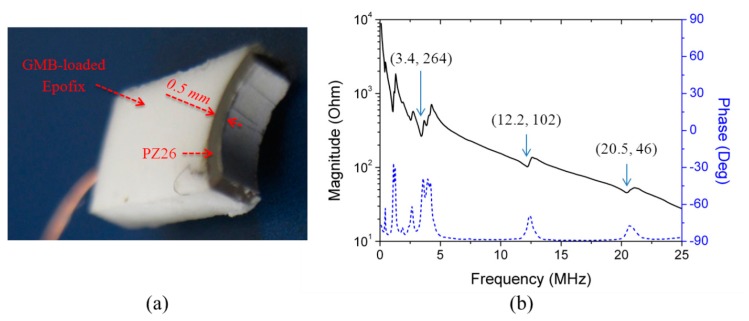
(**a**) A fabricated PZ26 quarter-ring focused transducer with GMB-loaded Epofix backing; and (**b**) its impedance spectrum in water.

A curved transducer was fabricated from a PZ26 ring (Ferroperm Piezoceramics A/S, Kvistgaard, Denmark) as shown in Figure 15a. GMB-loaded Epofix (mass ratio K1-GMB:Epofix = 1:9) was cast on the outer face to support the piezoelectric element. The height in the elevation direction was ∼1.5 mm, and the PZ26 thickness was ∼0.5 mm. The impedance spectrum of the transducer was measured in water, Figure 15b.

**Figure 16.**
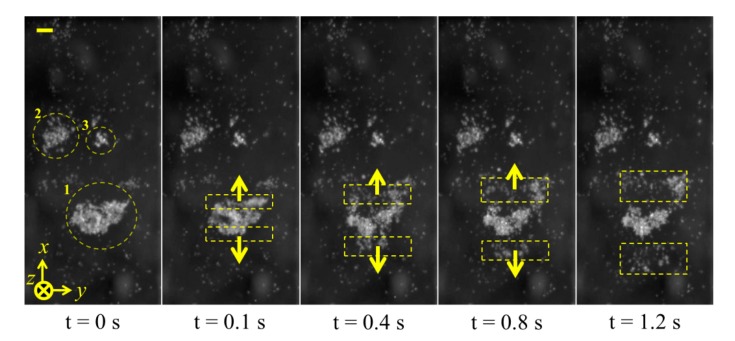
Image sequences of the effects of acoustic radiation forces acting on an agglomerate at the focal spot of the curved transducer. Scale bar = 100 μm.

**Figure 17.**
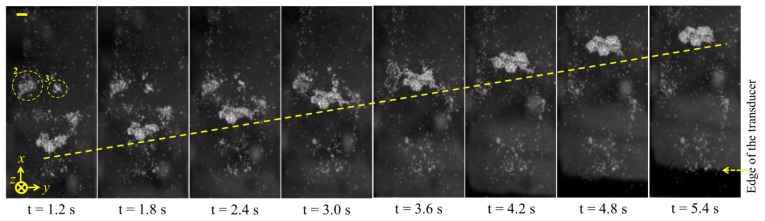
Image sequences of the transportation of the trapped agglomerate by moving the curved transducer in the direction of X-axis. Scale bar = 100 μm.

For experimental purposes, a small volume of Ø10 μm fluorescent polystyrene beads and degassed water (bead concentration ∼5 × 10^5^ particles/mL) was placed in a petri dish. The transducer was fixed on an XYZ stage and placed 2.5 mm away from the dish so that the transducer's focal point was located at the front surface of the glass. The polystyrene beads were allowed to settle, then the transducer was driven with a 10 V_pp_ CW sinusoidal signal at the fundamental resonance, *f* = 3.4 MHz. The motion of the particles was recorded at 10 fps. For detailed observation, the transducer was positioned above many large, settled particle agglomerates before it was turned on. As a typical observation, one of the particle agglomerates, positioned at the focal point of the transducer, denoted as 1 in Figure 16, rapidly divided into three parts because of acoustic radiation forces. The middle part of the agglomerate was concentrated, while the two side parts, highlighted in rings 2 and 3, were pushed away. These results correspond with the simulated velocity fields. After the middle agglomerate was trapped and concentrated, the transducer was moved at a constant speed in the positive *x*-axis direction, and the trapped agglomerate followed, Figure 17. During motion of the trapped agglomerate, the other settled agglomerates (denoted as 2 and 3 in Figure 17) were driven towards the trapping site, eventually forming a larger agglomerate.

**Figure 18.**
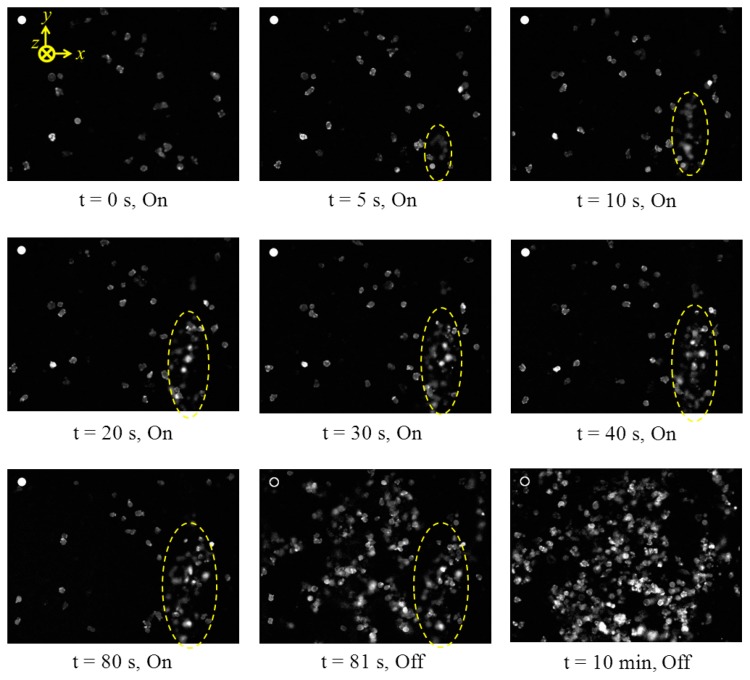
Image sequences of the formation of an agglomeration of cells in the curved ultrasonic resonator field. The circle at the top-left of each image is 10 μm diameter. The filled circle denotes that the transducer is activated.

The evaluation of the curved ultrasonic resonator in cell manipulation was also performed with Dicty cells. The device was driven with 8 V_pp_ CW sinusoidal signals at the 20.5 MHz 5th harmonic resonance of the transducer. Dicty cells at 10^6^ cells/mL concentration in KK2 buffer fluid were allowed to adhere to and migrate freely around a Petri dish. Images of the cells were captured every second during the experiment as shown in Figure 18. When the transducer was activated, an agglomeration of cells formed in suspension. The size of the agglomeration increased slowly in the first 30 s then stabilized, potentially because the number of cells in the volume was constant. During the application of the *F_PRF_*, the cells maintained their shapes and positions with no signs of ‘rounding up’, which is a characteristic of decreased cell vitality. When the transducer was deactivated, the agglomeration dropped onto the petri dish and the cells regained normal motility. It can also be seen that the agglomeration was formed mostly by suspended cells with few adhered cells on the petri dish. This is because the adherence forces of the cells are greater than the *F_PRF_* produced by the device at this voltage. This limit in acoustic force magnitude could be overcome with increased source voltages, albeit with the detrimental effect of increased heating and streaming [94]. This result of interaction between acoustic radiation forces and the Dicty cells suggests the curved ultrasonic resonator configuration has potential in measurement of the viscoelastic properties of biological cells and their adhesion forces to other objects.

### 4.3. 1-D Ultrasonic Array Manipulator

**Figure 19.**
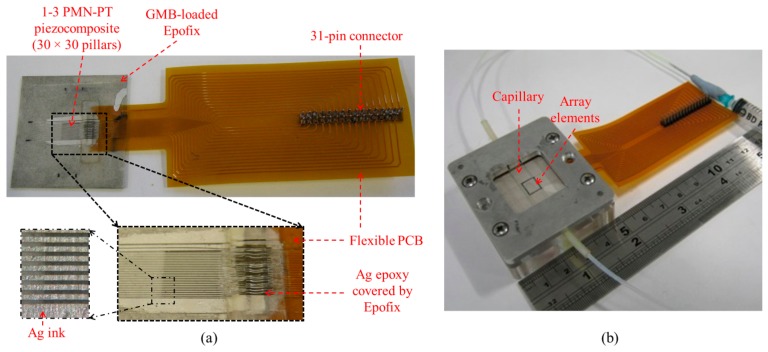
(**a**) 1-D 30-element ultrasonic array; and (**b**) complete device in a housing with a capillary channel.

A 1-D 30-element ultrasonic array was fabricated with 1-3 PMN-PT piezocomposite comprising 30 × 30 pillars cut in a 6 × 6 mm^2^ PMN-PT plate. The dimensions of each PMN-PT pillar were 100 × 100 × 650 μm^3^ and Epofix was used as the kerf filler. The piezocomposite was embedded in GMB‐loaded Epofix (mass ratio K1-GMB: Epofix = 1:9) and lapped to a thickness of ∼485 μm for a resonant frequency of 2.45 MHz, as shown in Figure 19a. A flexible PCB (fPCB, Flexible Dynamics, Glasgow, UK) with 30 signal tracks and a ground track was aligned to one edge of the piezocomposite and conductive Ag epoxy was used to connect the tracks to the edge of the piezocomposite. After the Ag epoxy cured, Ag ink was applied on both sides of the piezocomposite, ensuring good electrode connectivity. A thin layer of Epofix was moulded onto the Ag epoxy to protect it during the scratch‐dicing process with an 80 μm thick blade to obtain a 120 μm electrode width for the complete array shown with its PCB connector in Figure 19b. A rectangular glass capillary (W3530, VitroCom) with 6 × 0.3 mm^2^ internal cross section was centered over the array elements, coupled to them with medical US couplant (Diagnostic Sonar Ltd., Livingston, UK) and the two components were clamped together in a mechanical housing, as shown in Figure 19b.

**Figure 20.**
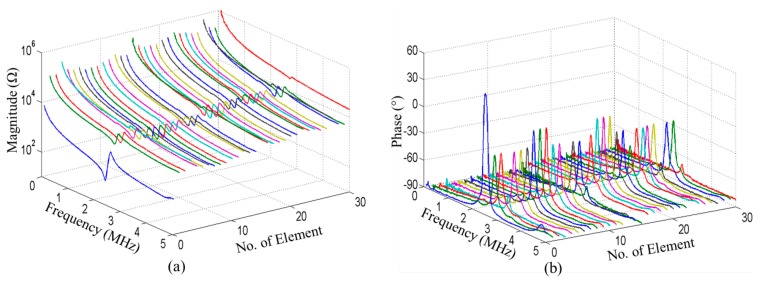
Impedance (**a**) magnitude and (**b**) phase of the 1-3 PMN-PT piezocomposite (No.0) and all 30 elements of the 1-D array (No.1–No.30).

The electrical impedance spectrum for each element was measured in air. The impedance magnitude and phase of the entire piezocomposite and all 30 individual elements of the 1-D array are shown in Figure 20. No other resonant mode was found around the fundamental resonance and relatively good uniformity of the elements has been achieved, although Elements 3, 15, 27 and 30 show somewhat higher impedance magnitude and lower impedance phase than the others. This may have been caused by degradation of the Ag epoxy connections to these elements after scratch-dicing. The impedance magnitudes of most elements at their resonant frequencies are in the range 2–3 kΩ, this high value caused by the small effective volume of PMN-PT in each element. It is reduced to some extent if multiple adjacent elements are driven simultaneously and a further reduction may be achieved by additional electric impedance matching.

An in-house system based on field programmable gate array (FPGA) electronics [95] was used to control the activation of subsets of array elements during particle manipulation experiments. Subsets of three adjacent elements of the array were activated together with a 17 V_pp_ CW sinusoidal signal at 2.55 MHz. A mixture of Ø10 μm green-fluorescent polystyrene beads in water (bead concentration ∼2.7 × 10^5^ particles/mL) was used. The fluorescent beads were observed and their motion in the capillary was recorded through an epi-fluorescence microscope. When the driving signal was applied, the beads moved quickly (>1 mm/s) to the pressure node and agglomerated above the common center of the active elements. The maximum lateral velocity of a single bead during the formation of bead agglomerates was measured to be ∼21 μm/s, corresponding to a lateral *F_PRF_* of 1.98 pN, calculated from Stokes' drag force. This is about 30% higher than for the 1-D array manipulator based on bulk PZ26 [34]. Taking into account the low effective volume of the piezomaterial and the mismatching electrical impedance in the active subset of the arrays, the PMN-PT piezocomposite has shown much better performance than bulk PZ26 in this application.

**Figure 21.**
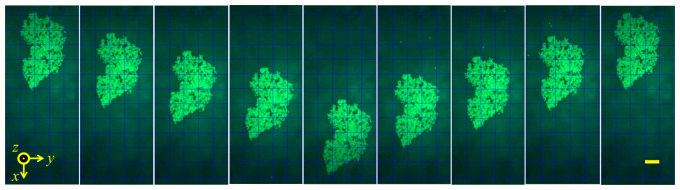
An agglomerate formed by activating three adjacent elements of the array is transported along the length of the capillary channel by altering the activated elements with a step size of one element. Scale bar = 100 μm.

After the agglomerate formed, the subset of three elements was altered along the array in one element steps. Figure 21 shows an agglomerate formed by three adjacent elements then transported along the length of the capillary channel in this way. During this process, no leakage of beads from the agglomerate was observed, indicating that they were strongly trapped by both *F_PRF_* and *F_SRF_*. It can be seen that the agglomerate length in the *x*-direction is about 500 μm, the same as the effective width of three adjacent elements. However, during transportation, the step size of the agglomerate is in the range 150–200 μm, not uniform. Also, the shift in the y-direction is up to 50 μm. These effects are thought to be due to the inhomogeneity of the US field caused by the differences in array elements and geometrical variation of the capillary channel, as well as misalignment between the capillary and the array elements, e.g., being not exactly perpendicular to each other.

**Figure 22.**
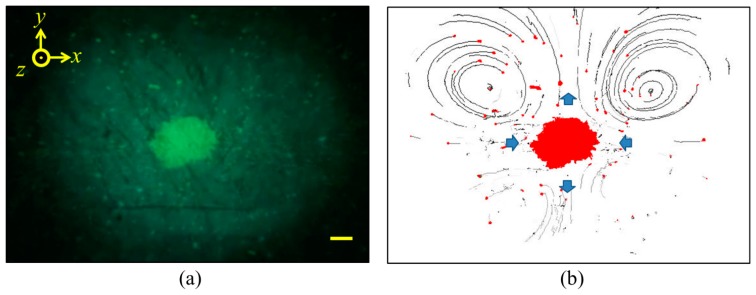
A four-quadrant acoustic streaming pattern occurs during formation of bead agglomerate: (**a**) image showing the original positions of the agglomerate and other beads (bright green); and (**b**) motion trajectory of the beads (red) affected by streaming. A set of three elements was driven at 20 V_pp_. Scale bar = 100 μm.

Streaming was observed during the formation of the agglomerates, Figure 22, but the pattern was different from the Rayleigh and Eckart types and related to the rotational component of active acoustic intensity caused by the capillary geometry [96]. The streaming had a four-quadrant pattern with the circulation planes parallel to the transducer plane (*xy*-plane) and the beads driven rapidly towards the agglomerate (blue arrows on the *x*-axis) by the combined effects of this streaming and lateral acoustic radiation forces. Some of the particles became part of the agglomerate, and the remaining fraction was driven away from the agglomerate (blue arrows on the y-axis) then affected by the side walls of the capillary, forming elliptical paths.

### 4.4. 2-D Matrix Thick Film Array Manipulator

**Figure 23.**
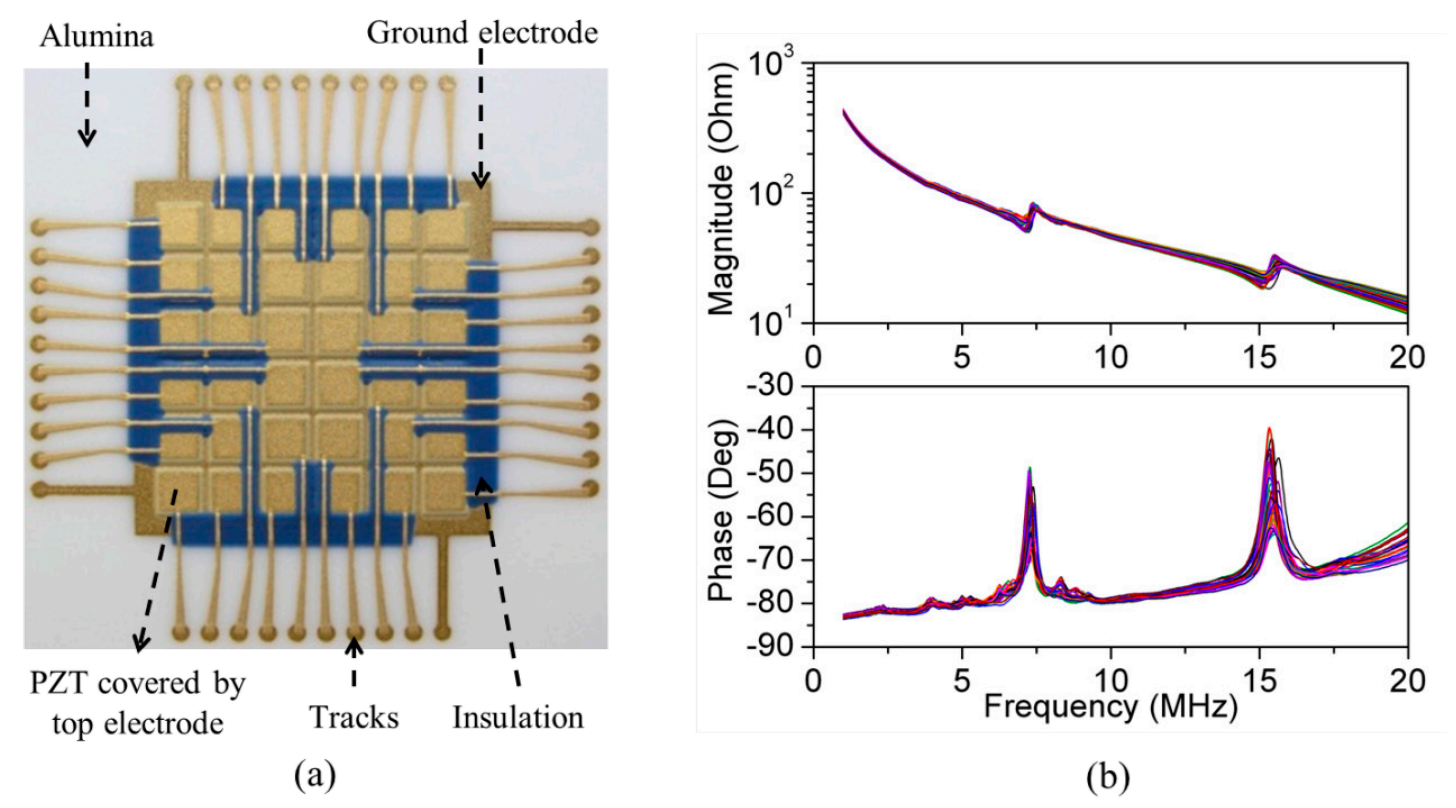
(**a**) PZT thick film 2-D matrix array; and (**b**) measured electrical impedance spectra of all 36 elements.

The final device was a PZT thick film 2-D matrix array with the dimensions of the 3-D FEA model fabricated with screen printing of a series of patterned thick films sintered on an Al_2_O_3_ substrate in a sequence including all functional layers from bottom to top, *i.e.*, the bottom Au electrode, piezoelectric thick films, top Au electrode, dielectric insulation layer and Au electrode fan-out tracks. The detailed fabrication process is reported elsewhere [91]. The array, Figure 23b, was poled with a 20 kV/cm DC electric field for one minute at room temperature. The residual porosity of PZT produced with this process is usually less than 10% [97]. The measured dielectric constant and loss are *ε_33_^T^*/*ε_0_* = 2250 ± 100 and *tanδ* = 0.09 ± 0.005 at 10 kHz, respectively [91]. The measured impedance spectra of all 36 elements of the array, Figure 23b, show that good uniformity of the array elements has been achieved with screen-printing technique, though with small variations introduced by the different configurations of the dielectric insulation layer and electrode interconnection layer near the edge of the PZT elements within the array. However, because more layers will be added to the bare array to form a resonant structure and the resonance is determined primarily by the thickness of the water layer, these small variations will have a limited effect in operation.

**Figure 24.**
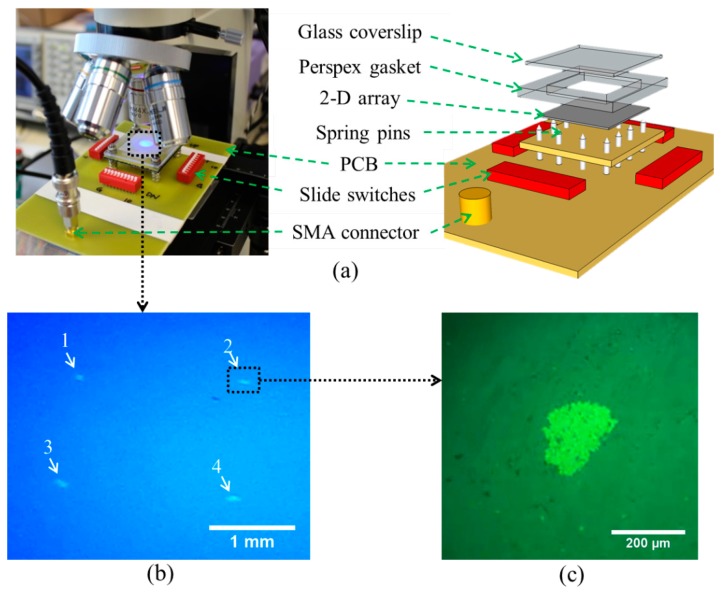
Trapping with four elements: (**a**) experimental set-up and schematic diagram of device; (**b**) photograph of four trapped agglomerates; and (**c**) microscope image of a trapped agglomerate.

The experimental set-up for this array is shown in Figure 24a. It was coupled to a fluid layer and a reflector with the bottom side of the alumina substrate to form a multilayer resonant structure. The chamber was formed with a Perspex gasket 2 mm thick and a glass slide 0.1 mm thick as a reflector, giving a water layer thickness of ∼10*λ_w_*. The measured resonant frequency of the whole arrangement was 7.26 MHz. The four central elements were driven with a 7.6 V_pp_ CW sinusoid at the resonant frequency. When the elements were activated, four agglomerates were formed and trapped in the chamber, as shown in Figure 24b. A corresponding result was achieved with all 36 elements but the radiation forces were smaller, took longer to form agglomerates, and moved fewer beads to the trapping sites. This is because of the additional electrical impedance mismatch caused by the parallel connection.

The results of particle manipulation demonstrate that the screen-printed PZT array can generate sufficient acoustic power to agglomerate beads. Also, as expected, the relatively large element dimensions resulted in a lack of geometrical overlap of energy gradients between adjacent elements. Therefore it was not possible to transport agglomerates laterally by switching activated array elements. The solution is to reduce the element size of array and to activate multiple elements together during operation, using the technique demonstrated in the 1-D ultrasonic array manipulator. This may also improve precision, because activation of multiple elements and alteration in one element steps smoothes lateral manipulation.

## 5. Conclusions

With the motivations to overcome the limitations of USM devices in terms of: (1) forces that can be generated; (2) constrained trapping sites due to the enclosure resonances and geometry of the devices; and (3) fabrication complexities negatively affecting progress towards mass production and commercialization, several multilayer resonator type USM devices have been investigated through simulation and experiment.

According to theory, high acoustic radiation forces can be achieved by increasing operating frequency and acoustic pressure gradients. Simulation of the planar ultrasonic resonator suggested that a HFUS transducer (>20 MHz) can give sufficient pressure gradients to achieve high *F_PRF_*; however, sensitivity to operating frequency brought difficulty to achieve these experimentally. The weak acoustic radiation forces generated in the current prototype device, however, still somewhat constrained cell motility. Thus, it is expected that stronger effects on motile cells can be realized by generating higher acoustic radiation forces. The curved ultrasonic resonator has shown a corresponding ability to manipulate microparticles and cells with increased acoustic radiation force through its utilization of focused US. In addition, trapped microparticles or cells could also be moved laterally with the assistance of motion stages controlling the transducer position. The results presented here suggest that quasi-USWs formed by a focused US transducer and reflector have potential in evaluation of the viscoelastic properties of biological cells and their adhesion forces to other objects.

The feasibility of precise lateral control of microparticles in a microfluidic channel has been demonstrated with US transducer arrays integrated into multilayer resonators. Thereby, the restriction that conventional USM devices can manipulate particles only in the direction of wave propagation has been overcome with controllable variation of acoustic energy distributions created by the transducer array. In this concept, 2-D (vertical and lateral) particle manipulation has been realized with a 1-D transducer array integrated into a multilayer resonator operating at ∼2.5 MHz; potentially 3-D (vertical, lateral and orthogonal) particle manipulation can be realized with a 2-D matrix array integrated into a multilayer resonator. Moreover, the use of 1-3 PMN-PT piezocomposite has given much better performance than conventional bulk PZT devices.

The development of the 2-D matrix thick film array manipulator has taken advantage of the relatively simple fabrication and interconnection strategy offered by screen-printing. Experimental results show good uniformity and acceptable film quality and performance have been achieved. Furthermore, the excellent reproducibility of screen-printing gives a potential for bulk production of disposable devices, benefiting the commercialization of Sonotweezers® for life sciences applications.

## Figures and Tables

**Table 1.** Outline capabilities of important contactless micromanipulation techniques.

		MT [4,11,14–17]	DEP [16,18–22]	OT [16,23–28]	USM [7,16,29–41]
**Particle sizes**	**<1 μm**	Yes	Poor	Yes	Poor
	**1–10 μm**	Yes	Yes	Yes	Yes
	**10–50 μm**	Yes	Yes	Poor	Yes
	**>50 μm**	Yes	No	No	Yes

**Typical force scale**		pN–nN	pN	fN–pN	pN–nN

**Particle preparation**		Yes	No	No	No

**Handling large numbers of particles**		Yes	Yes	Poor	Yes

**Handling individual particles**		Yes	Yes	Yes	Poor

**Basis of particle contrast**		Magnetic susceptibility	Dielectric constant	Refractive index	Density and compressibility

**Range of acceptable media**		Wide	Poor	Wide	Wide

**Spatial precision**		Medium	Medium	High	Low

**Range of operating field**		Long	Short	Short	Long

**Biocompatibility**		Poor	Fair	Fair	Good

**Challenge of system integration**		Low	Low	High	Low

**Table 2.** Basic specifications of USM devices with corresponding requirements in the selection of piezoelectric materials.

Specifications of USM Devices	Requirements of Piezoelectric Materials
High frequency (>1 MHz) to reduce likelihood of cavitation and achieve satisfactory spatial distribution of targeted particles or cells	Compatible resonant frequency constant
High acoustic radiation forces generated with low voltage electronics	High electromechanical coupling coefficient, e.g., *k_t_*, and transmission coefficient, e.g., *d_33_*
Continuous wave (CW) or near-CW drive whilst maintaining a stable temperature for cells with minimal heating	Low dielectric loss and high mechanical quality factor, *Q_m_*
Electrical impedance matching between transducer and control electronics	Compatible dielectric constant according to detailed transducer design
Biocompatibility of materials	Surface treatment
Commercialisation friendly	High ease of fabrication and low cost
